# Structural basis for activation of Swi2/Snf2 ATPase RapA by RNA polymerase

**DOI:** 10.1093/nar/gkab744

**Published:** 2021-08-24

**Authors:** Wei Shi, Wei Zhou, Ming Chen, Yang Yang, Yangbo Hu, Bin Liu

**Affiliations:** Section of Transcription & Gene Regulation, The Hormel Institute, University of Minnesota, Austin, MN 55912, USA; State Key Laboratory of Virology, Wuhan Institute of Virology, Center for Biosafety Mega-Science, Chinese Academy of Sciences, Wuhan 430071, China; University of Chinese Academy of Sciences, Beijing 100049, China; State Key Laboratory of Virology, Wuhan Institute of Virology, Center for Biosafety Mega-Science, Chinese Academy of Sciences, Wuhan 430071, China; University of Chinese Academy of Sciences, Beijing 100049, China; Roy J. Carver Department of Biochemistry, Biophysics and Molecular Biology, Iowa State University, Ames, IA 50011, USA; State Key Laboratory of Virology, Wuhan Institute of Virology, Center for Biosafety Mega-Science, Chinese Academy of Sciences, Wuhan 430071, China; Section of Transcription & Gene Regulation, The Hormel Institute, University of Minnesota, Austin, MN 55912, USA

## Abstract

RapA is a bacterial RNA polymerase (RNAP)-associated Swi2/Snf2 ATPase that stimulates RNAP recycling. The ATPase activity of RapA is autoinhibited by its N-terminal domain (NTD) but activated with RNAP bound. Here, we report a 3.4-Å cryo-EM structure of *Escherichia coli* RapA–RNAP elongation complex, in which the ATPase active site of RapA is structurally remodeled. In this process, the NTD of RapA is wedged open by RNAP β' zinc-binding domain (ZBD). In addition, RNAP β flap tip helix (FTH) forms extensive hydrophobic interactions with RapA ATPase core domains. Functional assay demonstrates that removing the ZBD or FTH of RNAP significantly impairs its ability to activate the ATPase activity of RapA. Our results provide the structural basis of RapA ATPase activation by RNAP, through the active site remodeling driven by the ZBD-buttressed large-scale opening of NTD and the direct interactions between FTH and ATPase core domains.

## INTRODUCTION

The Swi2/Snf2 family proteins regulate many nucleic acid transactions, such as DNA replication, transcription, DNA repair and recombination through mediating mobilization of various nucleic acid-protein complexes (1–4). The Swi2/Snf2 family members are classified into 24 subfamilies and one distinct RapA group based on sequence homology within the ATPase core, and the RapA group includes eubacterial and archaeal members with more variations in sequence than other subfamilies (5).

As the only bacterial homolog of Swi2/Snf2, RapA (also named HepA) is an abundant RNA polymerase (RNAP)-associated protein with ATPase activity (6,7). It binds to nucleic acids with a slight preference for DNA–DNA and DNA–RNA duplexes (8–10). RapA stably binds to the RNAP core enzyme, but not the holoenzyme due to its overlapping RNAP-binding site with sigma factors (7,11). RapA was suggested to use the energy of ATP hydrolysis to facilitate the release of stalled RNAP and promote RNAP recycling during transcription (5,10,12). The RapA deletion strains showed no significant alterations in their growth rates, mutation rates or UV sensitivity, suggesting that the role of RapA might differ from those proposed for eukaryotic Swi/Snf proteins (6). However, RapA deletion causes severe inhibition of bacterial growth under high salt concentrations (12).

RapA contains seven domains and three linker regions: N-terminal domain (NTD), two RecA-like domains 1A and 2A, two Swi2/Snf2-specific domains 1B and 2B, Spacer domain, and C-terminal domain (CTD), and Linkers 1 to 3. The two RecA-like domains (1A and 2A) and two Swi2/Snf2-specific domains (1B and 2B) form the ATPase core of RapA, a characteristic module of the Swi2/Snf2 proteins (11). Within the Swi2/Snf2 family, the ATPase core domain is typically accompanied by auxiliary domains, which presumably regulate the ATPase activity in a fashion of modular allostery (13). The formation of an activated ATPase structure relies upon displacement of an auxiliary domain (14), whose role is played by the NTD in RapA. RapA NTD makes contacts with the conserved ATPase core in the crystal structure of RapA (11). It was reported that the ATPase activity of RapA is autoinhibited by its NTD, as the NTD deletion enhances the ATPase activity (15), suggesting the NTD may allosterically regulate the ATPase activity of RapA. Additionally, the ATPase activity of RapA is stimulated by RNAP in an allosteric fashion (6), which is supported by the small-angle x-ray scattering experiments (15).

Our previous 4.7-Å resolution crystal structure of RapA–RNAP complex (16) shows a direct interaction between RNAP and RapA, but does not reveal the molecular details of this interaction or potential structural changes in RapA upon RNAP binding. In addition, the NTD of RapA has a poor density and makes contacts with the symmetric mates, suggesting the architecture of the complex may be affected by crystal packing. Therefore, to understand how RNAP stimulates RapA’s ATPase activity by inducing its conformational change, we used single-particle cryo-electron microscopy (cryo-EM) to determine the structure of RapA–RNAP elongation complex.

The 3.4-Å resolution cryo-EM structure determined in this study reveals the detailed interactions between RNAP and RapA and shows a structural remodeling of ATPase active site in RapA, which is driven by the RNAP β' zinc-binding domain (ZBD)-buttressed large-scale opening of NTD and the extensive hydrophobic interactions between β flap tip helix (FTH) and ATPase core 1A/2A domains. Further functional tests demonstrated that both β' ZBD and β FTH are essential for activating the ATPase activity of RapA. Our findings provide new insights into the action mode of how the ATPase activity of RapA is stimulated by RNAP, which might also shed light on the regulatory mechanism of other Swi2/Snf2 ATPase proteins.

## MATERIALS AND METHODS

### Preparation and assembly of RapA–RNAP elongation complex

The *Escherichia coli* RNAP core enzyme and the N-terminal His-tagged RapA protein were overexpressed and purified separately as previously described (11,17–19). The strains, plasmids and oligos used in this study are listed in Supplementary Table S2. The purified RNAP core enzyme was mixed with an excess of RapA and loaded onto a Superdex G200 prep grade gel filtration column (GE Healthcare) equilibrated in the buffer (20 mM Tris–HCl pH 7.5, 50 mM NaCl, 0.1 mM EDTA, 5 mM MgCl_2_). The fractions containing the RapA–RNAP complex were pooled and concentrated to around 20 mg·ml^−1^. The synthetic DNA scaffold was prepared by annealing the Nontemplate DNA to an equal molar amount of Template DNA at 95°C for 10 min and cooling to the room temperature slowly. The DNA/RNA hybrid used in the assembly was prepared by mixing the annealed DNA scaffold with RNA09 (1:1 molar ratio) at 80°C for 10 min and then slowly cooling to the room temperature. The RapA–RNAP elongation complex was assembled by directly incubating 10 μM RapA–RNAP with 25 μM DNA/RNA hybrid in a buffer containing 20 mM Tris–HCl, pH 7.5, 50 mM NaCl, 5 mM MgCl_2_ and 0.2 mM AMPPNP at 37°C for 10 min.

### Cryo-EM grid preparation and data acquisition

The final concentration of 8 mM CHAPSO was added and mixed in the reaction mixture of RapA–RNAP elongation complex immediately before grid preparation. A drop of 4 μl of the complex was applied to Quantifoil R2/2 200-mesh Cu grids (EM Sciences) glow‐discharged at 15 mA for 60 s. Grids were blotted for 4 s at 22°C under the condition of 100% chamber humidity and plunge-frozen in liquid ethane using a Vitrobot Mark IV (FEI). The grids were imaged using a 300 keV Titan Krios microscope equipped with a Falcon III direct electron detector (FEI) at the Hormel Institute, University of Minnesota. Data were collected in the counting mode with a pixel size of 0.89 Å and a defocus range from −1.0 to −2.4 μm using EPU (FEI). Each micrograph consists of 32 dose-framed fractions and was recorded with a dose rate of 0.95 e^−^/pixel/s (1.25 e^−^/Å^2^/s). Each fraction was exposed for 1 second, resulting in a total exposure time of 32 s and the total dose of 40 e^−^/Å^2^.

### Image processing

Cryo-EM data were processed using cryoSPARC v2.15 (20), and the procedure is outlined in Supplementary Figure S1. A total of 3218 movies were collected. Beam-induced motion and mechanical drift were corrected with dose-weighting using the Patch motion correction (21). The contrast transfer functions (CTFs) of the summed micrographs were determined using Patch CTF estimation (22). Particles were then automatically picked using Blob picker with the parameters: minimum particle diameter (110 Å) and maximum particle diameter (210 Å). In total, 1 456 920 particles were picked with a 384 pixels of box size. Junk particles were removed through three rounds of 2D classifications. The 121 225 particles from the good 2D classes were used for Ab-initio Reconstruction of four maps. The initial models were low pass filtered to 20 Å and set as the starting references for heterogeneous refinement (3D classification) in cryoSPARC v2.15. A total of 58 761 particles in the correct 3D class were selected to perform homogeneous refinement and non-uniform refinement successively, generating the final 3.4 Å map for our RapA–RNAP complex. Resolutions of the maps were determined by gold-standard Fourier shell correlation (FSC) at 0.143 between the two half-maps. Local resolution variation was estimated from the half-maps by ResMap (23).

### Model building and refinement

The initial model was generated by docking the previous structures of the components in the RNAP core (PDB ID: 6B6H) and RapA (PDB ID: 6BOG) into the individual cryo-EM density maps using Chimera (24) and COOT (25). The 3.4 Å cryo-EM density maps for RapA–RNAP complex allowed us to build the NTD domain of RapA and the DNA/RNA hybrid in COOT. The upstream DNA part was not built in the model due to poor density. The intact model was then refined using Phenix (26). In the real-space refinement, minimization global, local grid search and adp were performed with the secondary structure, rotamer, and Ramachandran restrains applied throughout the entire refinement. Histogram and directional FSC plots for the cryo-EM map was analyzed and generated by 3DFSC web server, and 3DFSC calculation shows the cryo-EM map has a sphericity value of 0.980 (Supplementary Figure S1E), suggesting that the map has no directional resolution anisotropy issue (27). The split cryo-EM maps were generated using color zone with 3 Å coloring radius in volume viewer of Chimera (24). The final models have good stereochemistry by evaluation in MolProbity (28). Model-to-map validation was also evaluated in Phenix. The statistics of cryo-EM data collection, 3D reconstruction and model refinement were shown in Supplementary Table S1. All figures were generated using UCSF ChimeraX (version 1.0) (29). The surface conservation analysis of residues of RapA is performed using the ConSurf server (https://consurf.tau.ac.il/) (30,31).

### ATPase activity analysis

For functional test of RapA, RapA protein with C-terminal His-tag was expressed to avoid the potential influence of N-terminal His-tag. Briefly, the wild-type *rapA* or N-terminal 321 bp deleted gene (encoding wild-type or NTD-deleted RapA) was cloned into pET21a plasmid in fusion with C-terminal His-tag using a ClonExpress II One Step Cloning Kit (Vazyme). In a similar way, the clone expressing N-terminal His-tag fused with wild-type or NTD-deleted RapA was constructed based on pET28a plasmid by introducing a TEV cleavage linker. Deletion of FTH domain in RNAP was constructed by mutating the *rpoB* gene in pVS10-RNAP plasmid using overlap PCR. Mutated fragments were digested by *Nco* I and *Sbf* I, which were then inserted into the same digested pVS10-RNAP plasmid. The RNAP-ΔZBD protein was prepared using pVS10-RNAP-ΔZBD plasmid. Protein purification was performed as described previously (32). The N-terminal His-tag of RapA expressed by pET28a plasmid was cleaved by TEV protease (33).

The ATPase activity was detected by measuring the consumption of [α-^32^p]ATP and generation of [α-^32^p]ADP as described previously (15). Briefly, 10 μl reaction mixture containing 1 μl 10 × TB buffer (40 mM Tris–HCl, pH 7.8, 40 mM KCl, 5 mM MgCl_2_, 1 mM DTT, 50 μg/ml BSA), 1 μl 2 μM RapA, 1 μl 1 μM RNAP, 1 μl 1 mM ATP, 0.5 μCi [α-32p]ATP and appropriate nuclease-free water, was incubated at 37°C for 60 min. Then, 5 μl 5% SDS was added to stop the reaction. Afterwards, thin layer chromatography (TLC) analysis was used to separate [α-^32^p]ATP and [α-^32^p]ADP. For this purpose, 1 μl reaction solution was spotted on a polycellulose plate (Sigma) and was dried at room temperature for 20 min. The TLC plate was then developed in aqueous solution containing 1 M formic acid and 1 M LiCl. The phosphor screen (PerkinElmer) and the Cyclone Plus (PerkinElmer) were applied to detect the intensity of radioactivity. The amounts of ATP and ADP from three replicates were quantified by ImageJ software. Data are shown as mean ± SD from three experiments. Statistical analyses were performed using the unpaired Student's *t*-test (two-tailed) between each of two groups.

### Binding analysis between RapA and RNAP

The 500 μl of each reaction mixture, which contains 40 mM Tris–HCl (pH 7.9), 300 mM NaCl, 5% glycerol, 250 μg RNAP (where indicated) and 210 μg RapA (WT or NTD-deleted one), was incubated at 37°C for 30 min. Then, size exclusion chromatography (Superdex 200, GE Healthcare) was applied to separate the complex and separated proteins. Different fractions were collected and analyzed by 12% SDS-PAGE.

## RESULTS

### Overall structure of RapA–RNAP complex

**Figure 1.**
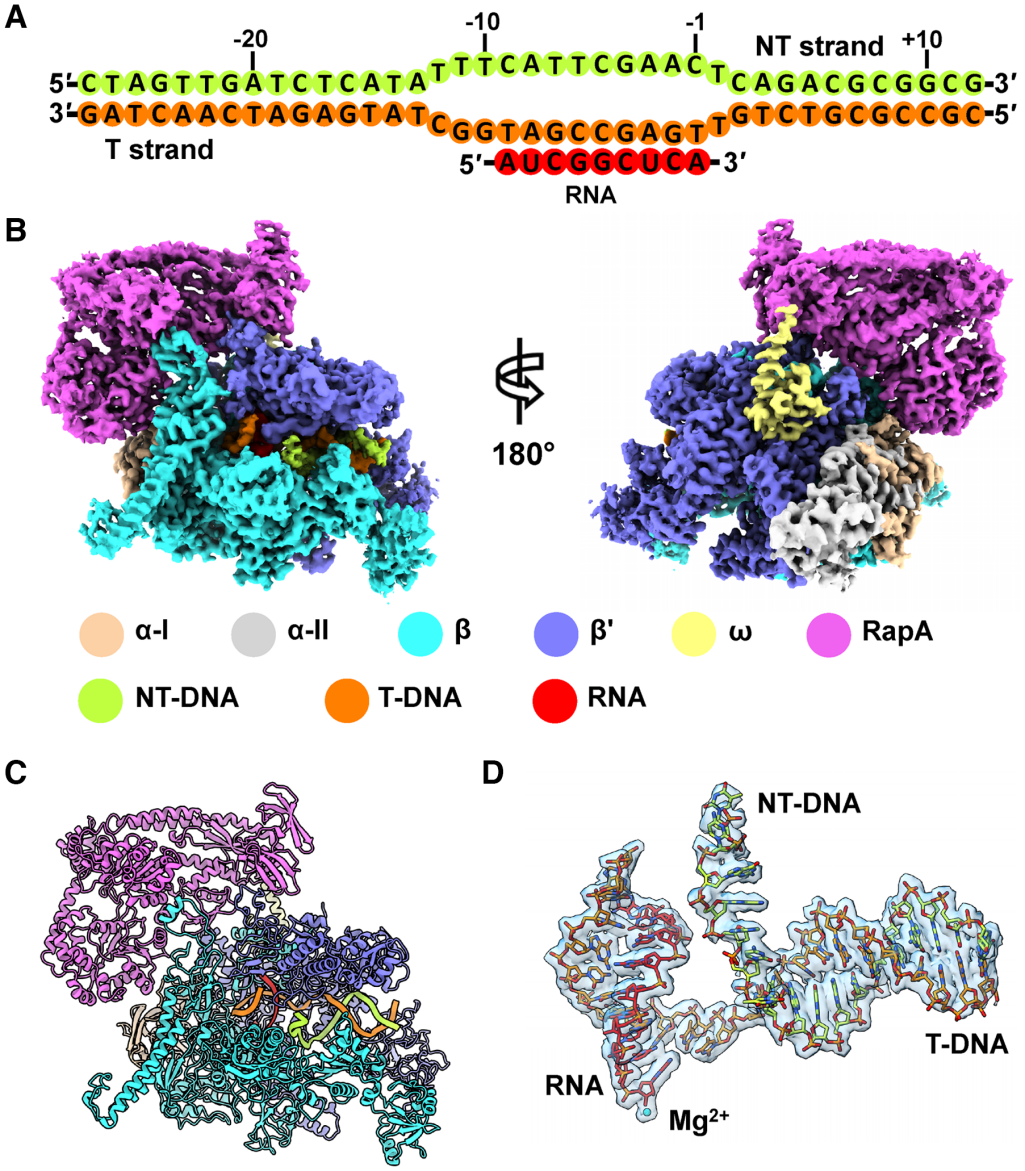
Cryo-EM structure of RapA–RNAP elongation complex. (**A**) Schematic representation of the nucleic-acid scaffold used for cryo-EM sample preparation. Green, nontemplate strand DNA; orange, template strand DNA; red, RNA. (**B**) Overviews of the cryo-EM map of *E. coli* RapA–RNAP elongation complex. The density maps are displayed in front and back views in surface representation. The color for each subunit is indicated. (**C**) The structural model of RapA–RNAP elongation complex. The subunit color code is the same as shown in (B). (**D**) Nucleic acid scaffold superimposed with the cryo-EM density contoured at level 0.2 in Chimera. The catalytic Mg^2+^ ion at the active site is shown as a cyan sphere.

To obtain the structure of *E. coli* RapA–RNAP elongation complex, we used a DNA–RNA scaffold (Figure 1A) similar to that used in previous study (34), aiming to increase the flexibility of the upstream DNA for RapA trapping. Using single-particle cryo-EM, we determined a 3.4-Å structure of the *E. coli* RNAP elongation complex bound to RapA (RapA–RNAP) (Figure 1B–C, Supplementary Figure S1 and Supplementary Table S1). The cryo-EM map shows well-defined density for all major components of the complex and supports reliable model building, except for the upstream DNA (Figure 1D and Supplementary Figure S2). The overall RapA–RNAP complex adopts essentially the same conformation and binding mode as reported previously (16). However, the relative positions of RapA to RNAP are slightly different in two structures, and the RapA–RNAP complex is more compact in the crystal structure than that in our cryo-EM structure (Supplementary Figure S3). Besides, the overall structure of our RapA–RNAP elongation complex is similar to that of the RapA–RNAP elongation complex (PDB ID: 7MKN) reported in a recent preprint manuscript (35), with a root-mean-square deviation (RMSD) of 2.3 Å (Cα aligned). In the preprint, the clamp in RapA–RNAP binary complex adopts a ‘closed’ state compared to that in the RNAP core enzyme (open clamp). Both RNAP elongation complex and RapA–RNAP elongation complex also show the ‘close’ state of clamp.

As reported previously (11), RapA in our complex has a modular organization and can be divided into several domains connected by three flexible linkers (Supplementary Figure S4). The NTD could be further divided into NTD-A and NTD-B subdomains, both of which fold as a bent antiparallel β sheet (Supplementary Figure S4B). The evolutionary conservation analysis of amino acids among 150 RapA homolog sequences shows that the 1A and 2A domains are highly conserved, indicating the functional importance of these two ATPase core domains, while other parts of the RapA are less conserved. In addition, the NTD-A subdomain is more conserved than the NTD-B subdomain (Supplementary Figure S4C).

### Structural remodeling of ATPase active site in RapA

**Figure 2.**
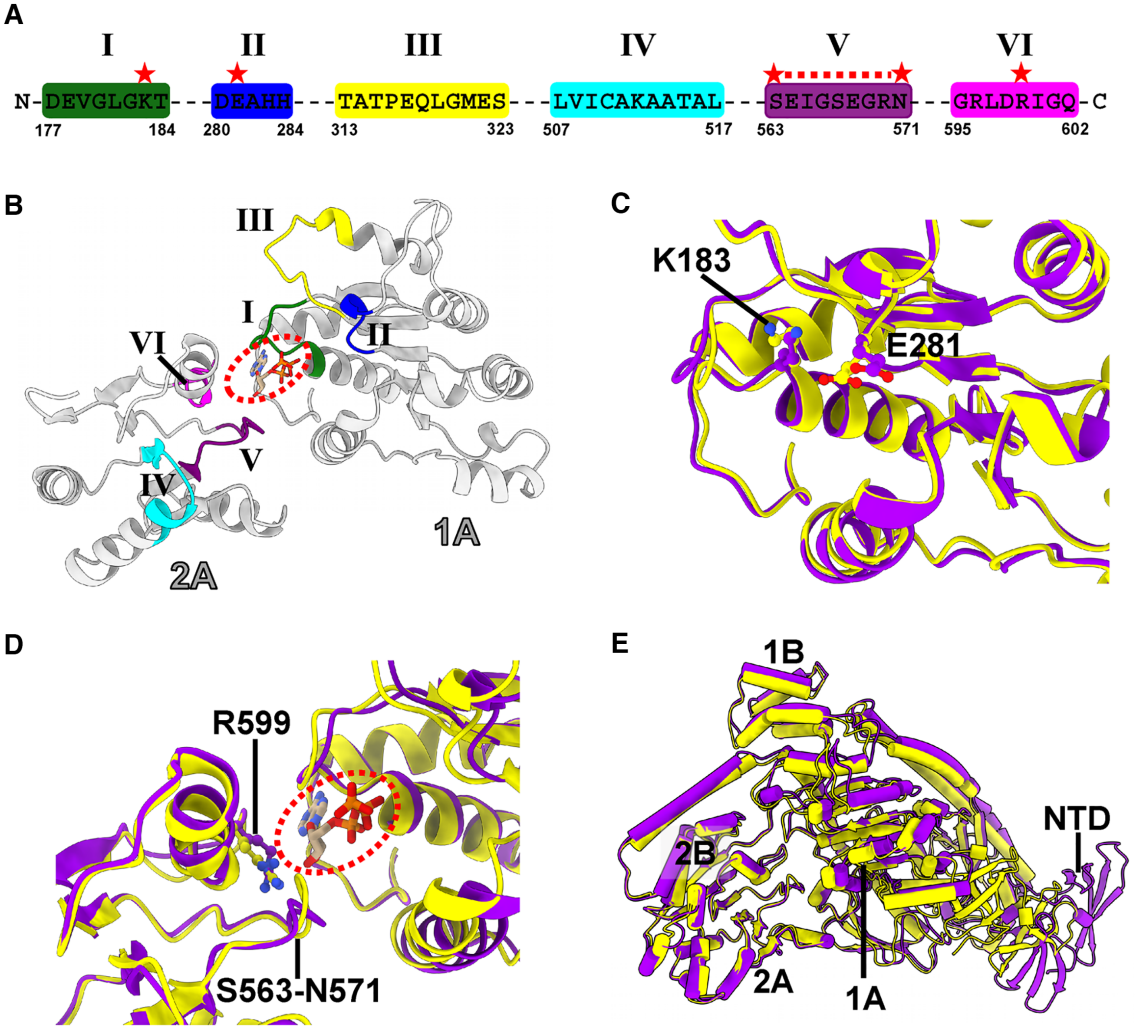
Conformational changes of ATPase motifs in RapA upon RNAP binding. (**A**) Sequences for the predicted six ATPase motifs in RapA. The critical residues, which are involved in the ATP binding or hydrolysis and exhibit conformational changes between apo and RNAP-bound RapA structures, are labeled with red stars. (**B**) Predicted structural motifs (Motifs I, II, III, IV, V and VI) locate in the 1A and 2A domains of RapA, forming the ATP-binding pocket. One ATP molecule (in dashed circle) is modeled in the binding site according to the superimposition of ATPase core domains of RapA with that of PcrA DNA helicase (PDB ID: 3PJR). The color code is as follows: Motif I (dark green); Motif II (blue); Motif III (yellow); Motif IV (cyan); Motif V (purple) and Motif VI (magenta). (**C**) Superimposition of the 1A domains between apo RapA (PDB ID: 6BOG, yellow) and RNAP-bound RapA (purple) structures. (**D**) Superimposition of apo RapA protein (PDB ID: 6BOG, yellow) and RapA in our RapA–RNAP complex (purple) using the 2A domains. The modeled ATP molecule is shown in the circled dash line. (**E**) Superimposition of apo RapA (PDB ID: 6BOG, yellow) and RNAP-bound RapA (purple) structures using the 2A and 2B domains. The alignment shows a ∼3 Å widening of ATPase active site cleft in our RapA–RNAP complex.

The ATPase core RecA-like domains 1A and 2A contain a set of conserved functional motifs (I–VI), which are predicted on the basis of helicase structures (11) (Figure 2A and B). Since AMPPNP is not observed in the ATPase active site, we have modeled one ATP molecule. Motifs I (Walker A), II (Walker B or DExx box), V and VI are in close proximity to the modeled ATP molecule in the active site (Figure 2B). Compared to the active site conformation in apo RapA structure (11), the active site in our RapA–RNAP complex adopts a different conformation, including the possible side chain rotamer changes of three conserved residues K183 (motif I), E281 (motif II) and R599 (motif VI) (Figure 2C, D and Supplementary Figure S5). All the three invariant residues are implicated to play critical roles in ATP and Mg^2+^ binding or ATP hydrolysis (36–38). Besides, the whole motif V (residues S563-N571) retracts from its position in the apo structure (∼1–4 Å) to provide a larger space in the active site (Figure 2D and Supplementary Figure S5B). In addition, when superimposing apo RapA structure (PDB ID: 6BOG) to our RapA–RNAP complex structure by the 2A and 2B domains, we observed a ∼3 Å distance of the ATPase core 1A and 1B domains farther away from 2A and 2B domains, widening the ATPase active site cleft of RapA (Figure 2E).

### Interactions between RapA and RNAP

**Figure 3.**
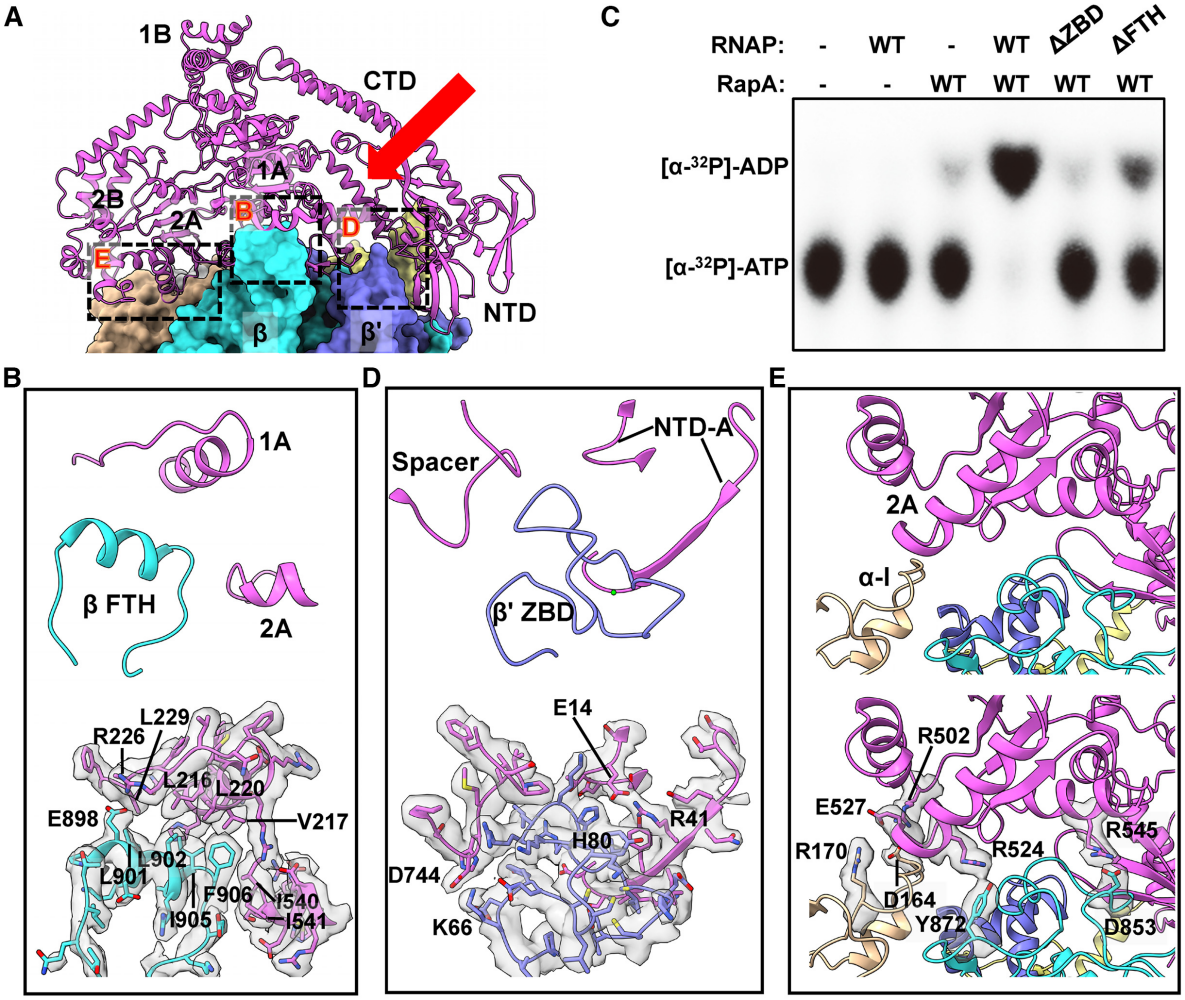
Interactions between RNAP and RapA. (**A**) Overview of RapA–RNAP interactions in the complex. RapA is shown as ribbons, and the RNAP subunits are shown in surface representation. The detailed interactions are shown for RapA with three regions of RNAP: β FTH (region B), β' ZBD (region D) and α and β subunits (region E), respectively. The red arrow indicates the position of the newly formed cleft due to the relocation of RapA NTD. (**B**) Close-up view of interactions of β FTH interactions with RapA in the dashed box area B. (**C**) Influence of deleting ZBD or FTH domains in RNAP on its activation to ATPase activity of RapA. The assays were repeated three times, and one representative result is shown. (**D**, **E**) Close-up view of interactions of β' ZBD or α/β subunits with RapA in the dashed box areas D and E. The residues involved in the interactions are indicated, and the densities of the corresponding residues are also shown in transparent surface representation. The color code for subunits is the same as in Figure 1.

The 3.4-Å RapA–RNAP complex structure reveals the detailed interactions between RapA and RNAP, mainly involving β FTH, β' ZBD, α subunit, and ω subunit of RNAP (Figure 3A). The structure of RNAP is not significantly altered upon RapA–RNAP interaction compared to the structure of RNAP elongation complex (PDB ID: 6XLM) (39), except for the β FTH. As reported previously (16), the association of RapA with RNAP causes a displacement of its β FTH motif, which is connected to the rest of the flap domain via flexible linkers and lies at the exit of the RNA channel. The β FTH motif interacts with 1A and 2A domains mainly through a large patch of hydrophobic interactions, involving residues L901, L902, I905, F906 of FTH and L216, V217, L220, L229, I540, I541 of RapA. Besides, the interface also includes a putative salt bridge interaction between β-E898 and RapA–R226 (Figure 3B). The β FTH motif interacts directly with both lobes (1A and 2A domains) of the ATPase core, which may contribute to the stuctural remodeling of the active site, affect the dynamics of RapA conformational changes during the ATP hydrolysis and thus enhance the ATPase activity of RapA. Consistent with this hypothesis, *in vitro* ATPase activity assay showed that while the wild-type RNAP activated the ATPase activity of RapA, deletion of the β FTH motif (residues 892–910) significantly decreased the activation of the ATPase activity of RapA (Figure 3C and Supplementary Figure S6). This decrease in ATPase activity is unlikely due to a weakened RapA–RNAP interaction, since the FTH-deleted RNAP core enzyme still shows strong binding to RapA (Supplementary Figure S7).

The β' ZBD contacts Spacer and NTD-A domains via two putative salt bridge interactions, β'-K66/RapA–D744 and β'-H80/RapA–E14 (Figure 3D). Interestingly, deletion of ZBD of RNAP core, which leads to only a slight decrease in its interaction with RapA (Supplementary Figure S7), almost abolished its ability to activate the ATPase activity of RapA (Figure 3C and Supplementary Figure S6). On the other side, the extensive interface between αI/β subunits and RapA 2A domain is supported by four putative salt bridge interactions, including αI-D164/RapA–R502, αI-R170/RapA–E527, β-D853/RapA–R545 and β-Y872/RapA–R524 (Figure 3E). In addition, the ω subunit may also play a role in supporting the binding between RapA and RNAP (Supplementary Figure S4D), but the local cryo-EM density does not support a reliable analysis of the detailed side-chain interactions.

### NTD of RapA undergoes a major conformational change

**Figure 4.**
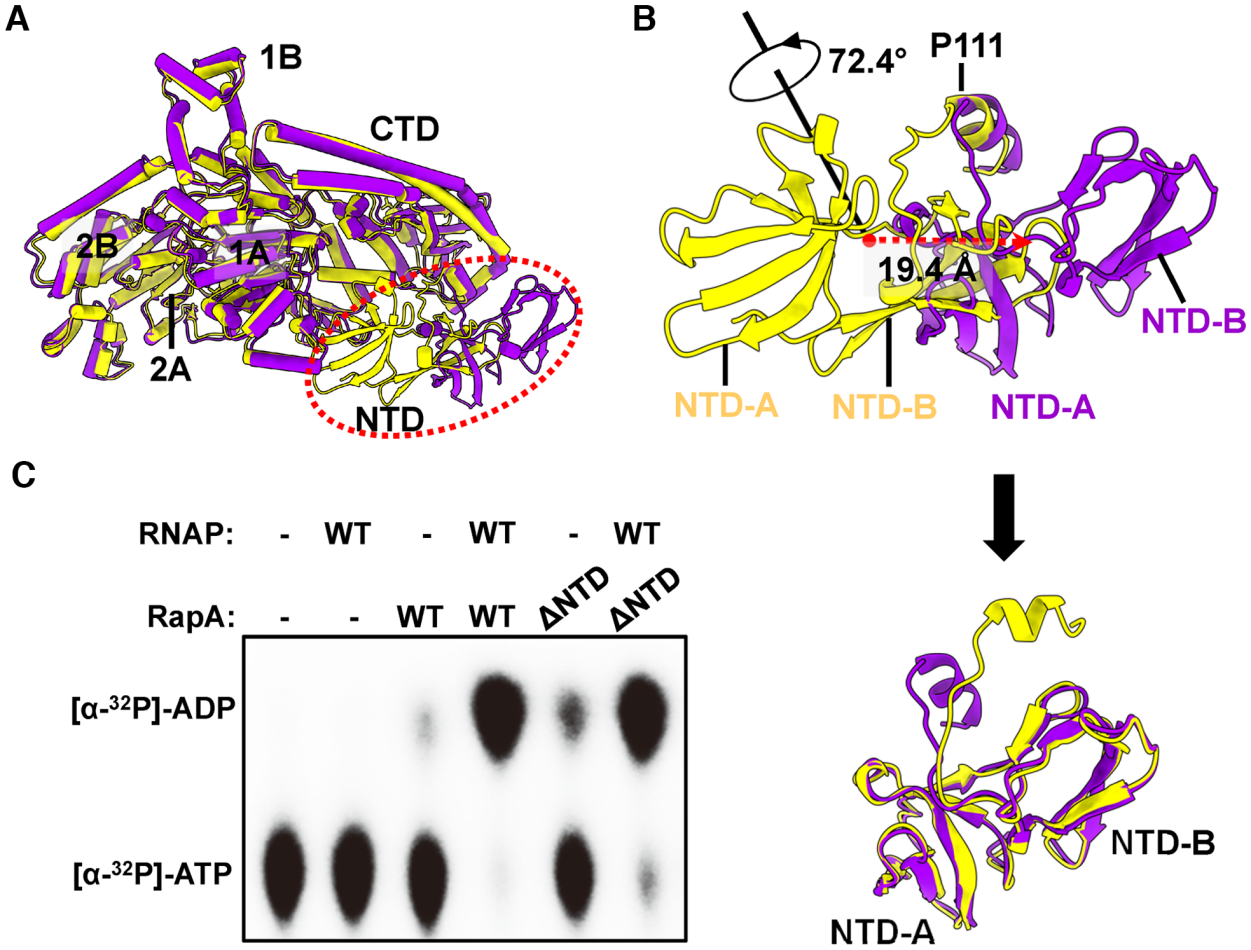
RapA NTD undergoes a major conformational change upon binding to RNAP. (**A**) Overall superimposition of RapA structures between apo RapA (PDB ID: 6BOG, yellow) and RNAP-bound RapA (purple), showing the NTD of RapA undergoes a dramatic movement upon binding to RNAP. The superimposition is performed using all Cα atoms in RapA. (**B**) The transformation needed to turn closed-NTD conformation in apo RapA into open-NTD conformation in the RapA–RNAP complex. The rotation axis and angle, and translation direction and distance are also indicated by arrows (Top). The superimposed NTD domains of apo RapA protein and RapA in our complex after the transformation procedure (Bottom). (**C**) Effects of RapA NTD on basal and RNAP-dependent ATPase activity of RapA. The assays were repeated three times, and one representative result is shown.

The structure of apo RapA protein adopts a closed-NTD conformation, in which the NTD makes contacts with the ATPase core 1A domain (11). While in our RapA–RNAP complex structure, the β' ZBD buttresses the RapA NTD and sterically keeps the NTD from reaching the 1A domain, which prevents the formation of a closed-NTD architecture in RapA (Figure 3A and D). This conformational change of NTD opening is also reported in the recent preprint manuscript (35). The whole RapA NTD domain (NTD-A and NTD-B) undergoes a significant rigid-body movement with a 72.4° rotation and 19.4 Å centroid translation away from 1A domain and adopts an open conformation (Figure 4A and B). Superimposition of the NTD alone from the apo RapA and RapA–RNAP complex structures shows a major difference in the position of the C-terminal α helix (Figure 4B). RapA P111 at the beginning of this small α helix may serve as a pivoting point as the neighboring loop region is relatively flexible (Figure 4B).

The opening of NTD leads to the formation of a new cleft between NTD, CTD and 1A domains (Figures 1B, C and 3A), and the role of the cleft remains to be studied. It was reported in the previous study that deletion of RapA NTD significantly activated its ATPase activity (15). However, we found that deletion of NTD (residues 1–107) only slightly increased the RapA’s ATPase activity in the absence of RNAP (Figure 4C and Supplementary Figure S6). This discrepancy might result from the extra N-terminal residues and His-tag constructed in the clone used in the previous study (15). To further confirm it, we expressed non-His-tagged RapA by the N-terminal His-tag cleveage using TEV protease and found that deletion of NTD in this non-His-tagged RapA also only slightly increased the ATPase activity in the absence of RNAP (Supplementary Figure S6).

Interestingly, RapA NTD deletion does not have a noticeable effect on either the RapA–RNAP interaction or RNAP-dependent activation of ATPase activity (Figure 4C, Supplementary Figures S6 and S7). Together with the evidence that ZBD-deleted RNAP is incapable of activating ATPase activity of RapA (Figure 3C), our results suggest that the opening of NTD, which is buttressed by β' ZBD, may play a critical role in RNAP-activated ATPase activity of RapA.

## DISCUSSION

### Proposed model for ATPase activation of RapA

In this work, we determined the cryo-EM structure of RapA–RNAP elongation complex, which reveals the detailed interactions between RNAP and RapA and the structural basis of ATPase activity stimulation by RNAP. We captured an open-NTD architecture of RapA buttressed by the RNAP β' ZBD and observed the extensive hydrophobic interactions between β FTH and RapA ATPase core domains. All the interactions and conformational changes provide basis of a structural remodeling in the active site of RapA. The RNAP-activated ATPase activity of RapA would probably promote the RNAP recycling during the transcription.

**Figure 5.**
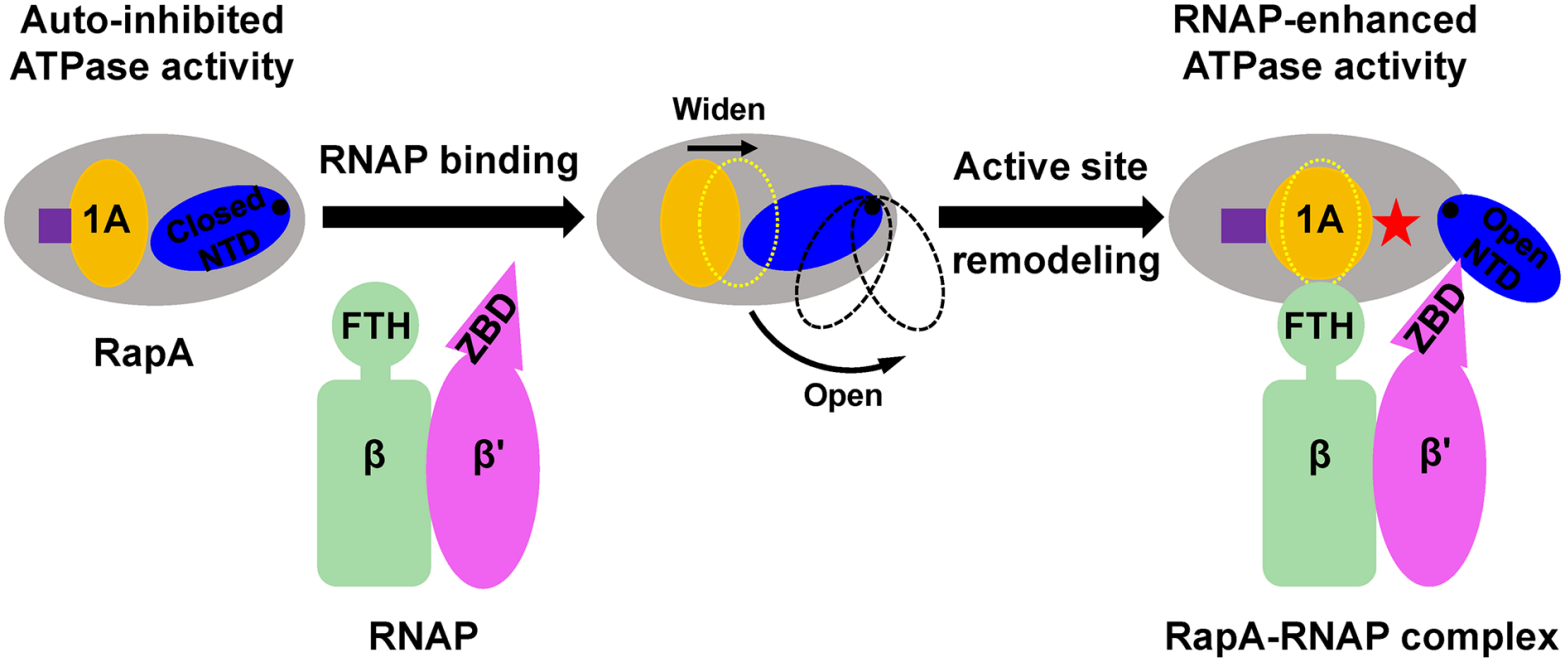
Proposed mechanism of RNAP-dependent activation of ATPase activity of RapA. The apo RapA exhibits a low ATPase activity due to the restriction of the ATPase core 1A domain by its closed NTD. Upon RNAP binding, the β' ZBD wedges between the 1A domain and NTD, buttressing an open NTD conformation and thus preventing NTD from contacting and restricting the ATPase core 1A domain. After the release from NTD opening, the 1A domain likely shifts away from the 2A domain in our complex compared to that in the apo RapA structure, significantly widening the ATPase active site cleft. Meanwhile, β FTH forms extensive hydrophobic interactions with ATPase 1A and 2A domains. All these interactions and conformational changes help to structurally remodel ATPase active site and stimulate ATPase activity. The ATPase active site is indicated in purple square or rectangle, and the active site remodeling is indicated by the shape change of yellow 1A domain. When the NTD of RapA is wedged open by β' ZBD in the complex, a novel cleft is formed on the other side, as indicated by a red star.

In the structure of apo RapA protein, the NTD tends to adopt a closed conformation, where NTD makes contacts with its ATPase core 1A domain (11). The restriction of 1A domain by NTD will likely impair its ATPase activity, since the conformational changes and dynamic response of ATPase core domains are significant for ATP hydrolysis cycle (40,41). This autoinhibited mechanism by the auxiliary domain could help avoid the unnecessary energy consumption in bacteria when no appropriate substrate or target binds to RapA. Upon binding to RNAP, RNAP β' ZBD domain inserts into the space between RapA NTD and 1A domains to open the NTD, which is critical for the activation of ATPase activity of RapA, as evidenced by the fact that ZBD-deleted RNAP lost its ability in activating ATPase activity of RapA (Figure 3C). It is presumed that the 1A domain is likely freed by NTD opening, as indicated by the superimposition result that the relative position of 1A and 1B domains moves ∼3 Å farther away from 2A and 2B domains compared to that in apo RapA protein (Figure 2E). The β FTH motif makes hydrophobic interactions with ATPase core 1A and 2A domains. Together with the release of NTD restriction to ATPase core domains (NTD opening) by ZBD, the β FTH-1A and β FTH-2A hydrophobic interactions may promote the structural remodeling in the active site of RapA and thus greatly enhance its ATPase activity (Figure 5). The active site remodeling possibly involves the conserved residues K183 (motif I), E281 (motif II), R599 (motif VI) and motif V. The three residues play critical roles in ATP and Mg^2+^ binding or ATP hydrolysis (36–38). The motif V (residues S563–N571) undergoes significant conformational change and opens up a larger space in the active site. The structural remodeling of ATPase active site might facilitate the dynamic process of ATP hydrolysis cycle. It should be noted that the open NTD state is not possible in the crystal lattice of the previous crystal structure of the RapA–RNAP complex (PDB ID: 4S20) due to the steric clashes between symmetry mates (16), suggesting the closed NTD state in the crystal structure likely resulted from crystal packing.

Although a non-hydrolysable ATP analog, AMPPNP, was included in the cryo-EM sample preparation, it is not observed in the active site of RapA in our complex structure. In the crystal structure of apo RapA, one sulfate ion occupied the proposed active site (11). It is possible that other substrates, such as DNA or RNA, is needed for stable ATP binding in the active site. Based on the superimposition of ATPase core domains with PcrA DNA helicase (PDB ID: 3PJR) (37), one ATP molecule is docked in the proposed ATP-binding site between the 1A and 2A domains of RapA (Figure 2B, D and Supplementary Figure S4B).

### β FTH and β' ZBD play distinct roles in different transcriptional steps

In this study, the RNAP β FTH motif forms extensive interactions with RapA, mainly through a hydrophobic patch on the short stretch. This hydrophobic patch was also shown to mediate the interactions between the β FTH and region 4 of σ^70^ to position the latter for recognition of the promoter –35 element (42,43). It was also reported that β FTH could block the RNA exit channel and interact with a nascent RNA stem-loop structure to inhibit transcription (44,45). Utilizing its hydrophobic patch, the β FTH may play multiple distinct and significant roles involved in various transcription steps.

We demonstrated a novel role of the β' ZBD of RNAP in regulating the ATPase activity of RapA. The β' ZBD was implicated to be involved in the protein-nucleic acid interactions of the transcriptional elongation complex (46) or the salt-resistant interaction with DNA (47). It was also reported that β' ZBD plays a role in transcription termination and antitermination (48–50). In a previous report, β' ZBD was found to help stabilize the upstream DNA and compensate for the weak σ4/−35 element interactions during transcription initiation (32). Besides, β' ZBD was shown to be involved in the interactions between RNAP and ribosome during their coupling (51,52). Here, we show that β' ZBD directly interacts with RapA NTD to activate its ATPase activity, indicating ZBD could play distinct roles in different functional processes.

### A newly formed cleft with potential function

Like other Swi2/Snf2 translocases with ATPase activity (53), the active site cleft between ATPase core domains (1A, 1B, 2A and 2B) of RapA is believed to bind upstream DNA. As the NTD is in the open conformation in our RapA–RNAP complex, another cleft is formed between the 1A domain, NTD and CTD on the opposite side (Figures 1B, C, 3A, 5 and Supplementary Figure S8). The newly formed cleft is likely involved in the transcription regulation, but the exact role of the new cleft needs further investigation.

In this study, we used the DNA/RNA hybrid with 9 base pairs to stabilize the transcription elongation complex. A longer single-stranded RNA was not used as we focused on the binding effect of the RNAP core, not the RNA transcript, on activating RapA’s ATPase activity. Although our structure did not trap the state of RapA binding to the upstream DNA or RNA and reveal the interactions between RapA and DNA/RNA, we have observed how RNAP core modulates RapA to enhance its ATPase activity in the structure and all the *in vitro* activity assays supported the observations, which only focused on RNAP, RapA and their mutants, while irrelevant to DNA or RNA. How DNA or RNA influences the structure and function of RapA–RNAP elongation complex will be elucidated in further studies.

In summary, we have determined the cryo-EM structure of RapA–RNAP elongation complex, which shows the detailed interactions between RapA and RNAP and the structural remodeling of ATPase active site of RapA by RNAP. The structure has revealed a novel open-NTD conformation of RapA buttressed by β' ZBD domain, and the extensive hydrophobic interactions between β FTH and ATPase 1A/2A domains. Our study provides insights into the mechanism of RapA ATPase activity stimulation by RNAP and sheds light on the regulatory mechanism of other Swi2/Snf2 ATPases, such as eukaryotic homologs with auxiliary domains. Besides, our RapA–RNAP complex structure could also benefit future studies on other aspects of RapA-mediated transcription regulation, such as directional translocation of DNA relative to RNAP.

## DATA AVAILABILITY

The cryo-EM density map for RapA–RNAP elongation complex reported in this paper has been deposited in Electron Microscopy Data Bank under accession number EMD-23716. The atomic coordinates for the atomic model are deposited in Protein Data Bank under the accession number 7M8E.

## Supplementary Material

gkab744_Supplemental_File
