# Supplementary material for: Structural basis for activation of Swi2/Snf2 ATPase RapA by RNA polymerase
Source: Nucleic Acids Res. 2021 Aug 24;49(18):10707–16. doi: 10.1093/nar/gkab744 (PMC8501970; doi:10.1093/nar/gkab744)
Supplement: gkab744_Supplemental_File [file gkab744_supplemental_file.pdf]

Supplementary Information for

Title: Structural basis for activation of Swi2/Snf2 ATPase RapA by RNA polymerase

Authors: Wei Shi[1,†], Wei Zhou[2,3,†], Ming Chen[2,3], Yang Yang[4], Yangbo Hu[2,*], Bin Liu[1,*]

Affiliations:

[1]Section of Transcription & Gene Regulation, The Hormel Institute, University of Minnesota, Austin, MN 55912, USA

[2]State Key Laboratory of Virology, Wuhan Institute of Virology, Center for Biosafety Mega-Science, Chinese Academy of Sciences, Wuhan 430071, China

[3]University of Chinese Academy of Sciences, Beijing 100049, China

[4]Roy J. Carver Department of Biochemistry, Biophysics and Molecular Biology, Iowa State University, Ames, IA 50011, USA

† The authors wish it to be known that, in their opinion, the first two authors should be regarded as Joint First Authors.

* To whom correspondence should be addressed. Tel: +1 507 437 9646; Email: liu00794@umn.edu

Correspondence may also be addressed to Yangbo Hu. Tel: +86 27 8719 9354; Email: ybhu@wh.iov.cn

Supplementary Figure S1

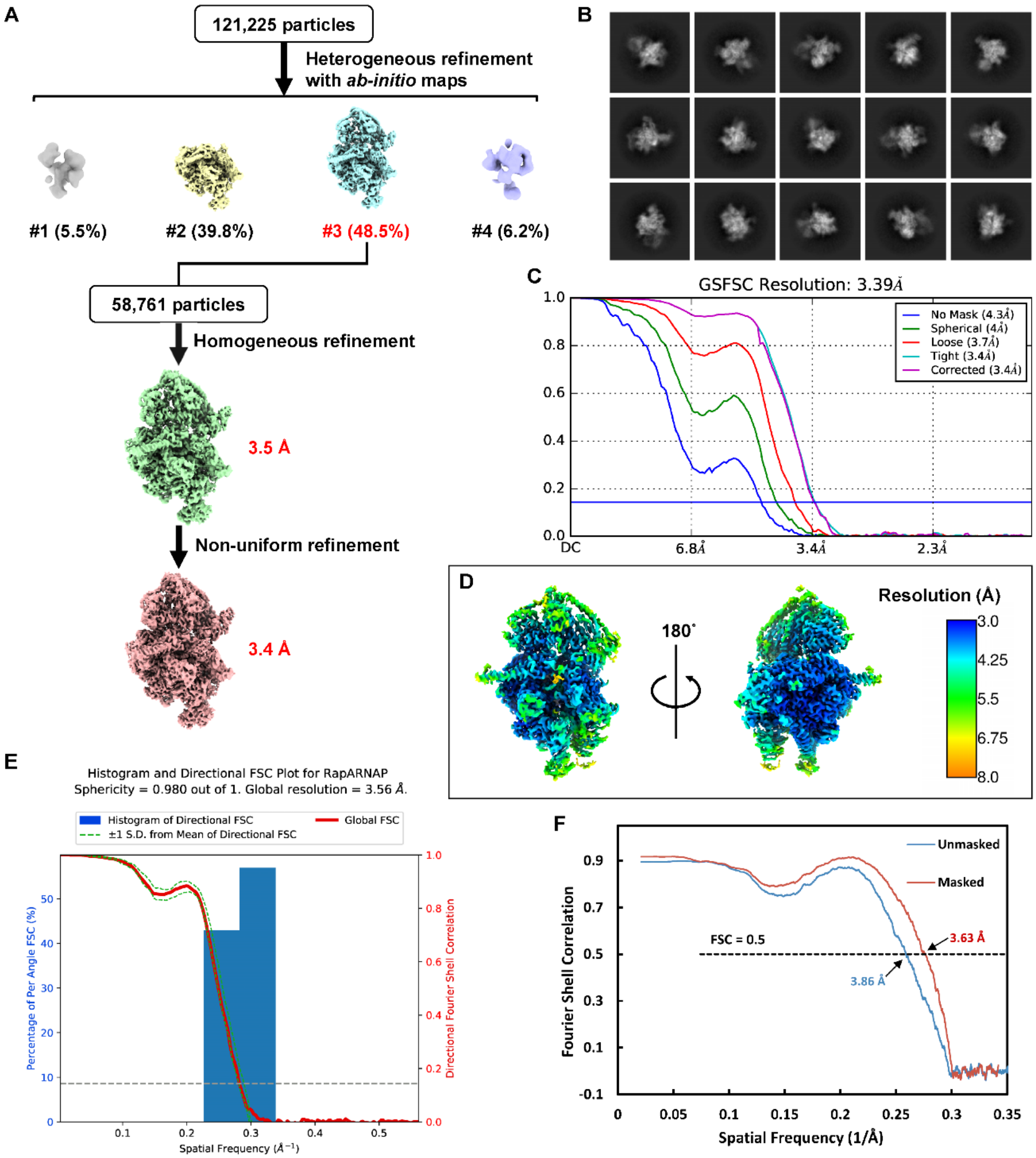

Supplementary Figure S1. Single-particle cryo-EM analysis of RapA-RNAP elongation complex. (A) Flow chart of cryo-EM data processing and map reconstruction for RapA-RNAP complex. **(B)** Representative 2D classes of RapA-RNAP complex from the final classification. **(C)** Gold-standard Fourier Shell Correlation (FSC) curves of the

final map. **(D)** The overall local resolution maps of the final 3D reconstruction. **(E)** Histogram and directional FSC plot for the cryo-EM map. The sphericity value of the map is also indicated. **(F)** FSC curves of model-to-map validated for the final 3D reconstruction.

Supplementary Figure S2

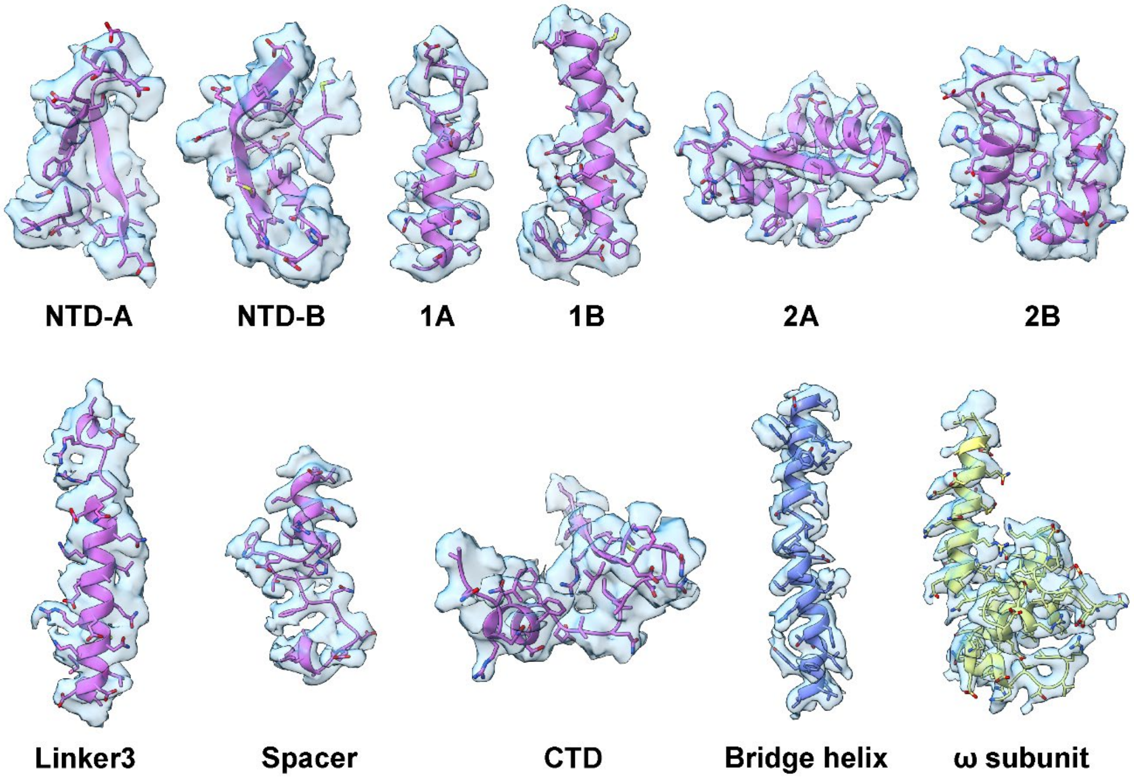

Supplementary Figure S2. Fitting of the atomic model and the 3D map in selected regions. Cryo-EM densities superimposed on the atomic models for representative regions of our RapA-RNAP complex. The cryo-EM densities were extracted using color zone with 3 Å radius and contoured at level 0.15 in ChimeraX.

Supplementary Figure S3

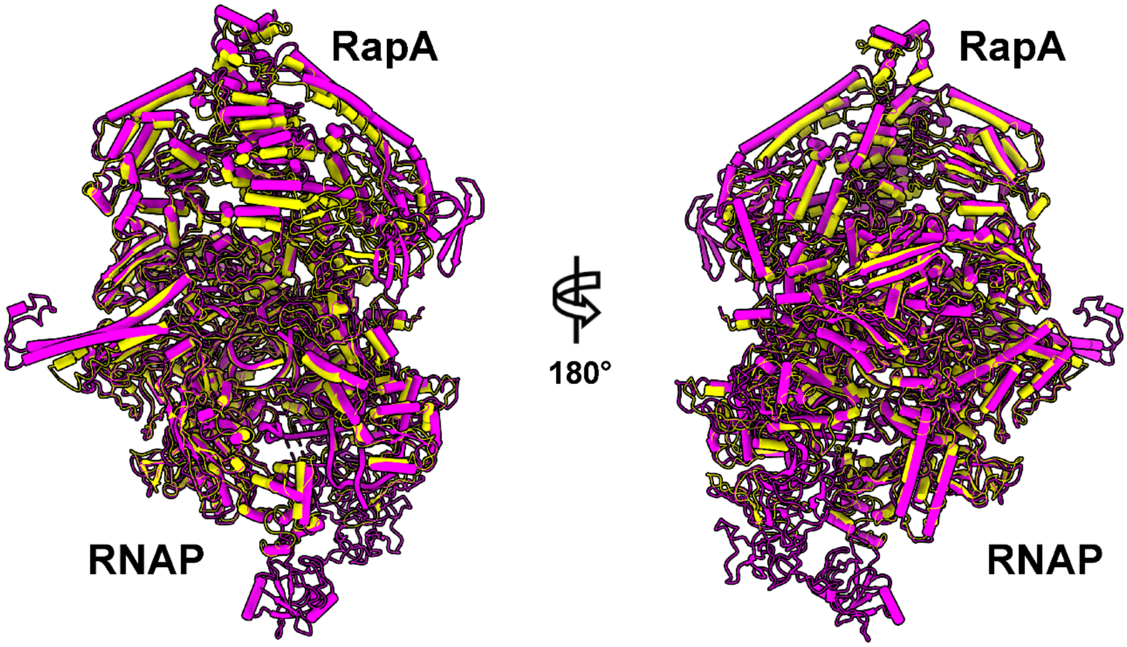

Supplementary Figure S3. Overall structural alignment of two RapA-RNAP complex structures. Superimposition of the crystal structure of RapA-RNAP (PDB ID 4S20, yellow) with our RapA-RNAP complex structure (magenta) using all Cα atoms from β and β' subunits, with a root-mean-square deviation (RMSD) of 1.8 Å (Cα aligned). Two views of the superimposition are displayed as a ribbon mode.

Supplementary Figure S4

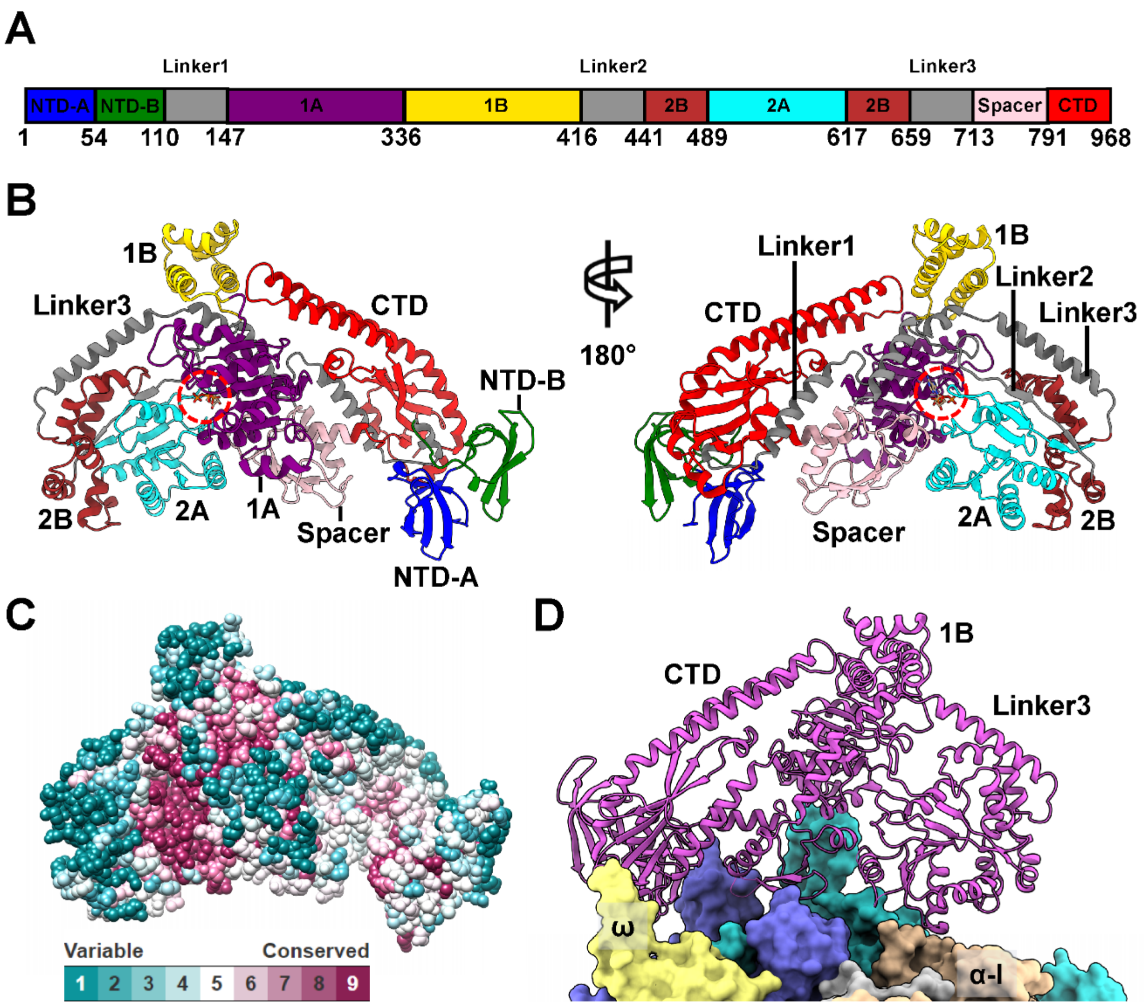

Supplementary Figure S4. Structural analysis of RapA in the complex. (A) Domain organization of RapA is shown with boundaries. The residue numbers in the sequence indicate the position of first residue of the following domain. Distinct domains are indicated and colored in different colors. **(B)** Structural organization of RapA domains in our RapA-RNAP complex. Two views of RapA structure are shown as a ribbon mode. The color code for domains is used as in A. One ATP molecule is also modeled at the supposed ATPase active site in the structure according to the superimposition of ATPase core domains with PcrA DNA helicase (PDB ID 3PJR). **(C)** The surface conservation

analysis of residues of RapA. The conservation degree is indicated as scores from 1 to 9, and number “9” indicates the most conserved and “1” indicates the most variable. **(D)** Back view of interactions between RapA and RNAP. The RapA protein is shown in ribbons, and the RNAP subunits are shown in surface representation. The color code for subunits is used as in Figure 1.

Supplementary Figure S5

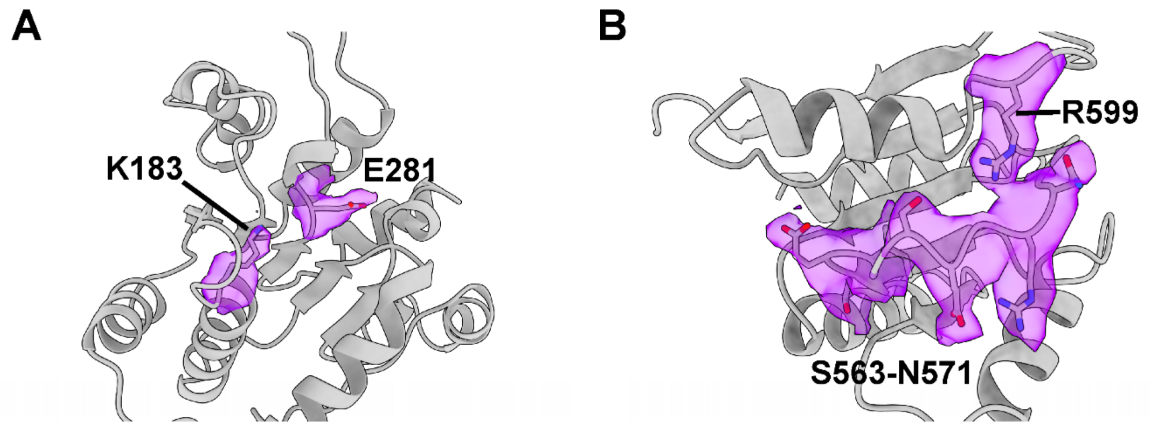

Supplementary Figure S5. Cryo-EM densities for the critical residues of ATPase active site of RapA. (A) The densities of residues K183 and E281 of RapA. **(B)** The densities of residue R599 and the whole motif V (residues S563-N571) of RapA. The cryo-EM densities are contoured at level 0.17 in Chimera.

Supplementary Figure S6

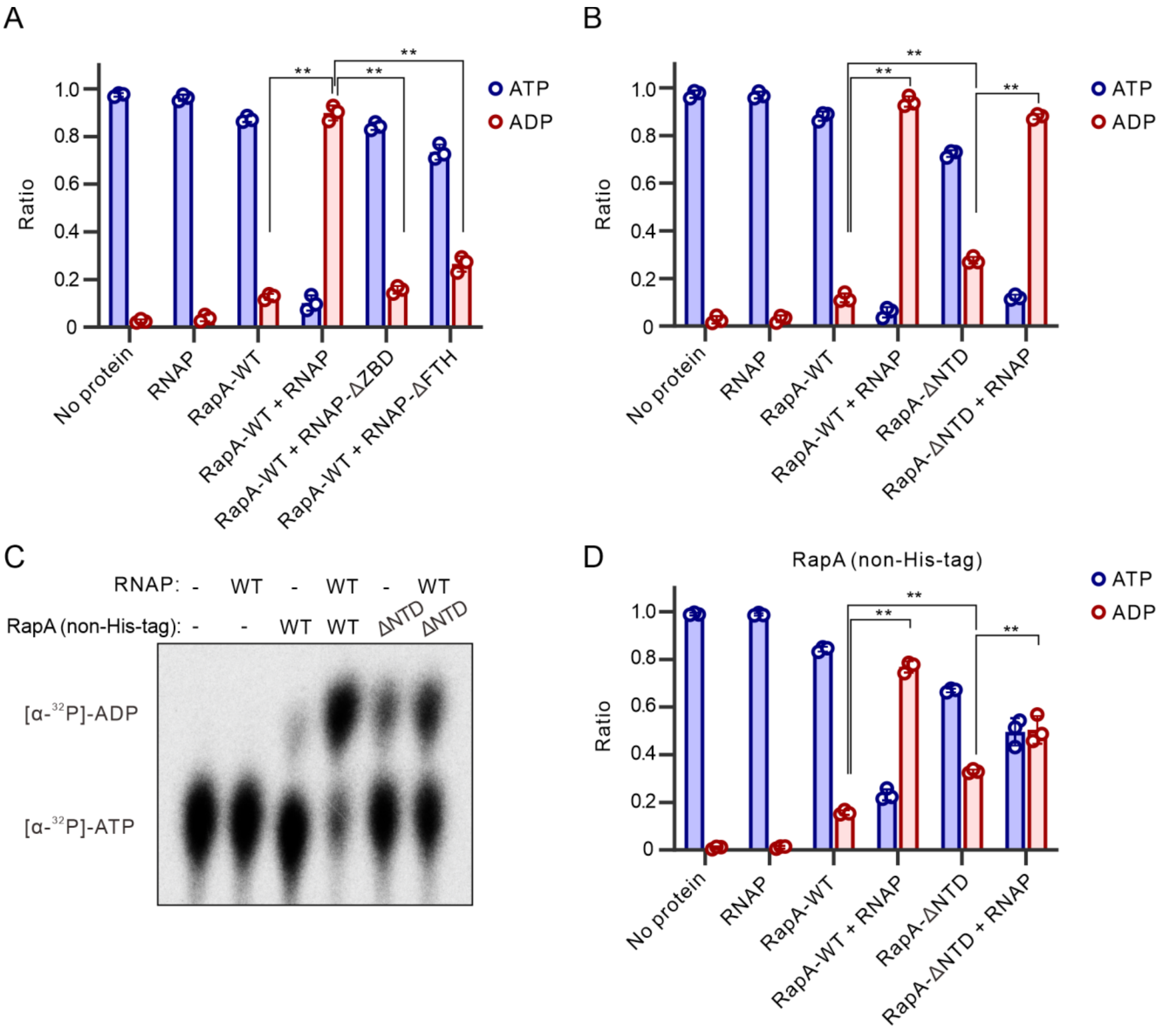

Supplementary Figure S6. The histogram of ATPase activity quantification analysis. **(A)** Influence of deleting the β' ZBD or β FTH motif in RNAP on its activation to the ATPase activity of RapA. **(B)** Role of the RapA NTD in ATPase activity without or with RNAP. (**C**) & (**D**) Effects of RapA NTD on basal and RNAP-dependent ATPase activity on non-His-tagged RapA. Y-axis value indicates the ratio of the quantity of the remnant ATP (blue column) or the yielded ADP (red column) to the total quantity of ATP and ADP. Experiments were repeated three times, and the quantification results are shown as

mean ± SD from three independent determinations. Statistical analyses were performed using the unpaired Student's t-test (two-tailed). ** $P < 0.01$.

Supplementary Figure S7

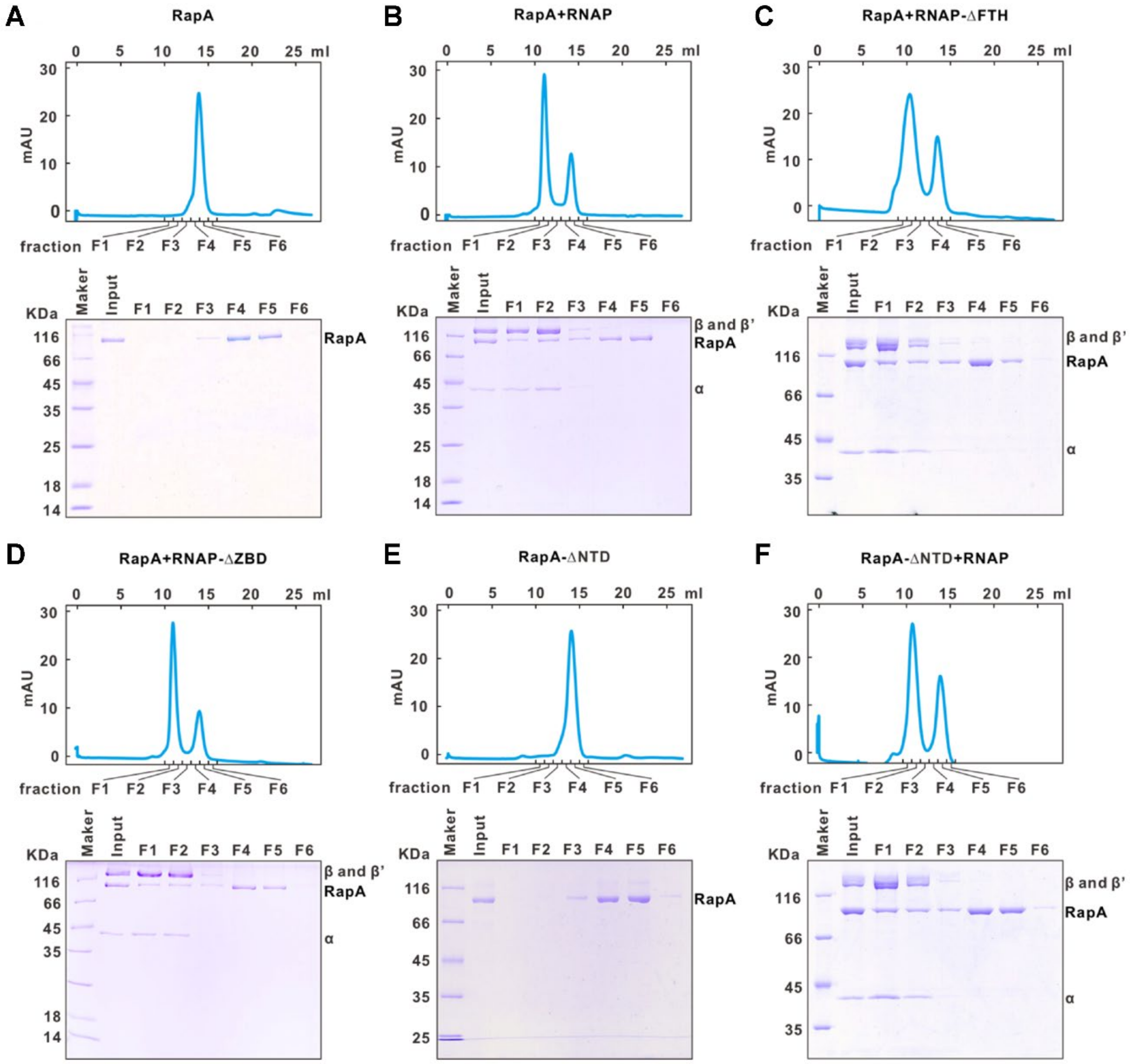

Supplementary Figure S7. The binding analysis between RapA and RNAP. Gel-filtration analysis of RapA-RNAP complexes: RapA **(A)**, RapA and RNAP **(B)**, RapA and RNAP-ΔFTH **(C)**, RapA and RNAP-ΔZBD **(D)**, RapA-ΔNTD **(E)**, and RapA-ΔNTD and RNAP **(F)**. Data for SDS-PAGE of different fractions in gel-filtration assays are shown at the bottom of each chromatogram panel.

Supplementary Figure S8

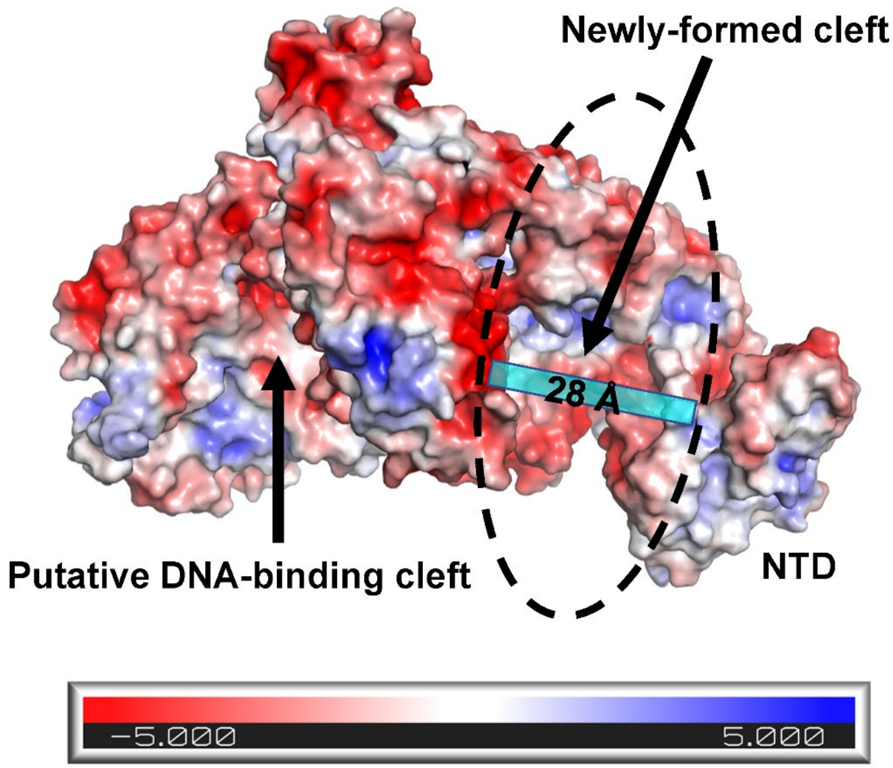

Supplementary Figure S8. Electrostatic surface potential of RapA. The newly-formed cleft (dotted black circle) and putative DNA-binding cleft are indicated by black arrows. The narrowest distance of the newly-formed cleft is around 28 Å. Electrostatic surface potentials were calculated by Adaptive Poisson-Boltzmann Solver (APBS) and contoured from –5 kT/e (red) to +5kT/e (blue).

Supplementary Table S1. Cryo-EM data collection, refinement and validation statistics.

**Data collection/processing**	**RapA-RNAP elongation complex**
Microscope	Krios
Voltage (kV)	300
Camera	Falcon III
Camera mode	Counting
Defocus range (μm)	−1.0 ~ −2.4
Electron exposure (e^-/Å^2)	40
Dose rate (e^-/pixel/s)	0.95
Magnified pixel size (Å)	0.89
**Reconstruction**	
Software	cryoSPARC v2.15
Symmetry	C1
Particles refined	58,761
Resolution (Å)	3.4
Map sharpening B-factor (Å^2)	-91.2
Access code	EMD-23716
**Model Statistics**	
Number of residues	4,230
Map CC	0.84
MolProbity score	2.14
All-atom Clashscore	11.49
Cβ outliers	0.00%
Rotamer outliers	0.00%
Ramachandran	
Outliers	0.07%
Allowed	10.64%
Favored	89.29%
RMS deviations	
Bond length	0.010
Bond angles	0.953
Access code	7M8E

Supplementary Table S2. Strains, plasmids and oligos used in this study.

Name	Application, or characters, or sequences	Source
Strains		
E. coli BL21(DE3)	Protein expression	Novagen
E. coli DH5α	Cloning construction	Shenzhen KT Life
Plasmids		
pVS10-RNAP	Plasmid expressing *E. coli* RNAP core enzyme, β′ subunit with C-terminal His_6-tag.	Addgene
pVS10-RNAP-ΔFTH	Plasmid expressing *E. coli* RNAP core enzyme, with deletion of β FTH.	This study
pVS10-RNAP-ΔZBD	Plasmid expressing *E. coli* RNAP core enzyme, with deletion of β′ ZBD.	Shi et al., *EMBO J*, 2020
pET21a-RapA	Plasmid expressing *E. coli* RapA protein, with C-terminal His_6-tag.	This study
pET21a-RapA-ΔNTD	Plasmid expressing *E. coli* RapA protein, with deletion of NTD (1-107 residues).	This study
pET28a-His-tev-RapA	Plasmid expressing *E. coli* RapA protein, with N-terminal His_6-tag and a TEV cleveage linker.	This study
pET28a-His-tev-RapA-ΔNTD	Plasmid expressing NTD deleted RapA protein (1-107 residues)), with N-terminal His_6-tag and a TEV cleveage linker.	This study
Oligos (5'-3')		
Nontemplate DNA	CTAGTTGATCTCATATTTCATTCGAACTCAGACGCGGCG	IDT, USA
Template DNA	CGCCGCGTCTGTTGAGCCGATGGCTATGAGATCAACTAG	IDT, USA
RNA09	AUCGGCUCA	IDT, USA
EcrpoB-F	GCAAGCATCGCTGACGAGTA	TIANYI, China
EcrpoB-d892-910-R	ACCTTTCGGCGTTACCTTACCAACCAGAATG	TIANYI, China
EcrpoB-d892-910-F	AGGTAACGCCGAAAGGTTCTGACGTTAAAGACTCTTCTCTGCGC	TIANYI, China
EcrpoB-R	GTACGACCGTTCACGTCATCAG	TIANYI, China
RapA-F	TAAGAAGGAGATATACATATGCCTTTTACACTTGGTCAACGC	TIANYI, China
RapA-dN107-F	TAAGAAGGAGATATACATATGTTCAGCAAACCGCAGGACCGTCT	TIANYI, China
RapA-R	GGTGGTGGTGGTGCTCGAGCTGATGCGTTACAACGATCAAACG	TIANYI, China
pET21a-F	CTCGAGCACCACCACCACCACCACT	TIANYI,

		China
pET21a-R	CATATGTATATCTCCTTCTTAAAGTTAAAC	TIANYI, China
RapA-28a-F	GAACCTGTACTTCCAATCCATGCCTTTTACACTTGGTCAACGC	TIANYI, China
RapA-dNTD-F	GAACCTGTACTTCCAATCCTTCAGCAAACCGCAGGACCGTCTG	TIANYI, China
RapA-28a-R	CTTCCTTTCGGGCTTTGTTATTACTGATGCGTTACAACGATCAAACG	TIANYI, China
pET28a-F	TAACAAAGCCCGAAAGGAAGCTGA	TIANYI, China
pET28a-R	GGATTGGAAGTACAGGTTCTCCATATGGCTGCCGCGCGGCACCAGGCCGCTG	TIANYI, China
